# Defining Parallels between the Salivary Glands and Pancreas to Better Understand Pancreatic Carcinogenesis

**DOI:** 10.3390/biomedicines8060178

**Published:** 2020-06-26

**Authors:** Céline Tiffon

**Affiliations:** 50 Rue des Vignes, 92000 Nanterre, Hauts-de-Seine, France; celine.tiffon@gmail.com; Tel.: +33-(0)689109162

**Keywords:** pancreatic cancer, salivary glands, diabetes mellitus, obesity, chemical pollutants, eating behavior

## Abstract

Pancreatic ductal adenocarcinoma (PDAC) is a highly malignant tumor with a dismal prognosis, largely due to its late presentation. Methods for early detection, the development of reliable screening tools, and the identification of sensitive and specific biomarkers have remained essential research priorities to improve early patient management and outcomes. The pancreas and salivary glands share histological and functional similarities, and the salivary glands have demonstrated a role in oral and systemic health. This review focuses on the similarities and differences between the pancreas and salivary glands and how these can inform our understanding of PDAC genesis and early diagnosis. In particular, chemical exposure, which alters salivary gland gene transcription and morphogenesis, may not only directly impact salivary gland regulation but alter pancreatic function via the systemic secretion of growth hormones. Diabetes and obesity are associated with an increased risk of pancreatic cancer, and a link between chemical exposure and the development of diabetes, obesity, and consequently PDAC genesis is proposed. Possible mechanisms include altering salivary or pancreatic morphology and organ function, disrupting endocrine signaling, or altering pro-inflammatory homeostasis. Finally, saliva contains putative specific biomarkers that show promise as non-invasive diagnostic tools for PDAC.

## 1. Introduction

Pancreatic cancers, of which >80% are classified as pancreatic ductal adenocarcinomas (PDACs) [1], are a lethal and devastating malignancy; only 2–9% of patients are alive within five years of diagnosis [2]. PDAC was ranked fourth in terms of tumor-related mortality in the Western world in 2015 [3]. This is at least in part due to its rapid spread in the asymptomatic phase of the disease [4]. One of the major difficulties with PDAC is its clinical silence; since the pancreas is located deep in the abdomen, the disease usually only presents after the tumor had already invaded the surrounding tissues or metastasized [4]. The consequence of late diagnosis is that only 15–20% of all patients have resectable tumors at diagnosis, and radical surgery still offers the only chance of cure, with very few effective adjuvant treatments currently available. PDAC is also complex and heterotypic; the desmoplastic reaction that occurs around cancer cells is considered a hallmark of PDAC, and the desmoplastic stroma, which contains high levels of cytokines and growth factors, is actively involved in tumor growth and dissemination and forms a barrier to chemotherapeutic drug delivery [1]. The incidence of PDAC has increased in recent years and is expected to continue to increase, surpassing breast and colon cancer to become the second leading cause of cancer-related deaths by 2030 [5]. In the past decade, overall cancer incidence in the USA increased by 21%, while, in comparison, PDAC incidence rose by 44%, with its lethality remaining very high, at approximately 85% [4]. The current standard of care for most patients with advanced PDAC is gemcitabine-based chemotherapy [6] including combinations with nab-paclitaxel or the aggressive chemotherapy regimen folinic acid, 5-fluorouracil (5-FU), irinotecan, and oxaliplatin (FOLFIRINOX), which provide a modest improvement in survival for patients with advanced disease [7,8]. Preventative measures are currently also limited; >80% of pancreatic cancers are multifactorial and due to sporadic mutations and <10% are due to inherited germline mutations [9]. The recognized non-hereditary risk factors include age (>55 years), pancreatitis, diabetes, tobacco smoking, excess body weight, alcohol abuse, dietary factors, and toxin exposure [10], but these do not completely explain disease risk [11,12,13,14].

As with other cancers, pancreatic cancer has both genetic and epigenetic origins, with the environment playing a role not only in individual risk but also risk in subsequent generations through parental transmission. Exposure to industrial pollutants is implicated in pancreatic carcinogenesis, with some of these chemicals shown to be mutagenic in several cancer types [15] or induce developmental defects in tissues such as the breast [16,17] or salivary glands [18]. To reduce the public health burden, there is a critical need to improve scientific knowledge on the causes of pancreatic cancer and to provide guidance for preventive measures. Additionally, early detection would provide the opportunity to improve patient outcomes and quality of life.

The pancreas and salivary glands have similar anatomical structures and physiological functions. The salivary glands are considered important in the regulation and maintenance of homeostasis [19,20], providing a rich source of biologically active peptides including epidermal growth factor (EGF). Gland dysfunction, which can be promoted either by disease or sialoadenectomy, modifies exocrine and endocrine secretion to disturb homeostasis. Alterations in salivary gland development in offspring have been shown to be associated with changes in salivary gland-specific gene expression and taste preferences after exposure to chemical pollutants such as endocrine disruptors. Therefore, given the similarities between the salivary glands and pancreas, it is reasonable to ask whether such exposure can affect pancreatic function. Interestingly, salivary gland function is impaired in diabetes mellitus, and deficiencies in salivary gland secretion of EGF may be a cause of some of the pathology associated with diabetes [20]. Furthermore, a link between the two organs prompts the question of whether human saliva may be a source of biomarkers for pancreatic adenocarcinoma, such that the salivary gland or its secretions, which are easy to access, could form the basis of future diagnostic tools. The salivary proteome could reflect the health status of an individual or their individual risk of developing a disease.

This article describes how similarities between the pancreas and salivary glands can help with our understanding of PDAC genesis and early diagnosis. Major risks factors for pancreatic cancer are discussed, especially the link between diabetes, obesity, and chemical pollutants, i.e., endocrine disruptors, exposure to which can affect tissue morphogenesis, hormone signaling, alter food satiety, and favor fat storage or disrupt pancreatic function. Obesity is an important modifiable factor for pancreatic cancer risk and is linked to increased mortality from PDAC and the promotion of desmoplasia through the action of various adipokines, among which leptin is a candidate that affects chemotherapy effectiveness due to its proinflammatory, proangiogenic, and survival effects [1].

## 2. Parallels with the Salivary Glands

### 2.1. Anatomical and Physiological Parallels

**Figure 1:**
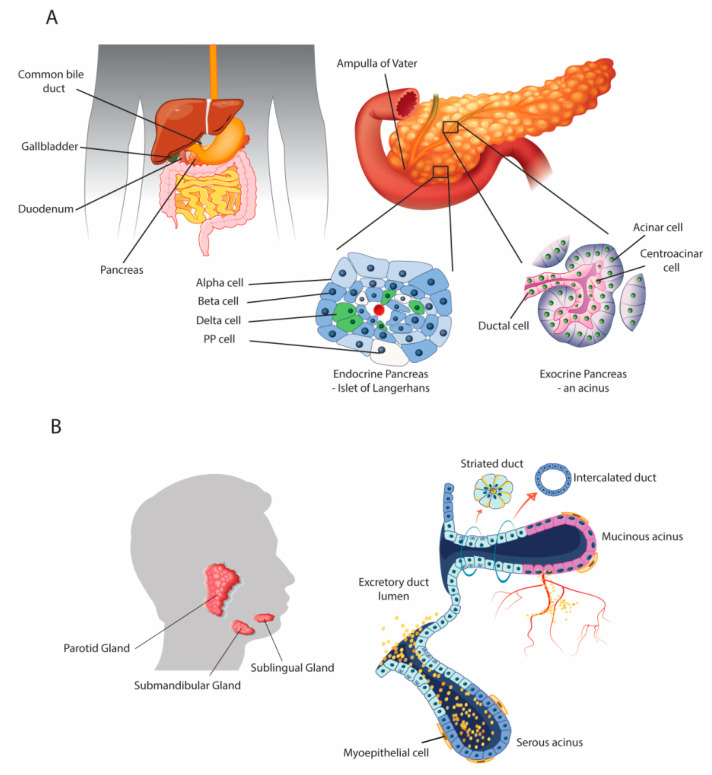
Anatomical structures of the pancreas and salivary glands. Both the pancreas and salivary glands are composed of exocrine acinar structures, and both have endocrine functions by secreting chemical messengers into the bloodstream to exert effects on distant organs. (**A**) The pancreas is a retroperitoneal organ connected to the liver proximally by the common bile duct and distally to the duodenum via the pancreatic duct. Exocrine pancreas acinar cells secrete pancreatic juice into the acinar ducts, which is eventually released into the duodenum via the ampulla of Vater to digest chyme. The endocrine pancreas is formed from islets of Langerhans distributed throughout the pancreas, which are formed predominantly of *α*- and *β*-cells, which are glucagon and insulin secreting, respectively, but also minor cell types including delta cells (somatostatin) and pancreatic polypeptide (PP) cells. (**B**) Similarly, the salivary glands (parotid, submandibular, and sublingual) are formed of acini, which secrete saliva from mucous or serous units via ducts. Saliva contains a complex bioactive mixture including digestive enzymes. Although not traditionally thought of as an endocrine organ, the salivary gland also secretes bioactive molecules (e.g., epidermal growth factor (EGF), hepatocyte growth factor (HGF) and transforming growth factor alpha (TGF-*α*)), into the bloodstream to affect distant organs.

The human digestive system comprises not only the gastrointestinal tract but also the accessory organs that aid digestion. The pancreas and salivary glands are exocrine and endocrine organs that share similar anatomical structures and physiological functions (Figure 1). Both glands are organized into acini and ducts [21], share analogous embryonic development involving epithelial-mesenchymal interactions, and produce fluids rich in bicarbonates containing digestive enzymes and other molecules to be delivered into the gut [21,22]. Moreover, similar mechanisms and processes are activated during the course of chronic disease in both organs. For example, alcohol misuse affects the pancreas and salivary glands in similar ways [23]. Furthermore, salivary and pancreatic progenitors are also very similar, including their role in repair and regeneration [24]. However, the two organs also differ in that they produce gland-specific secretions [21], while dissimilarities are also found regarding the inflammatory diseases that affect them, such as chronic pancreatitis, autoimmune pancreatitis, and Sjögren’s syndrome [25]. Gittes [24] presented a comprehensive review of all aspects of pancreatic development but did not explore the importance of differentiation of stem cells into mature pancreatic tissue or post-natal regeneration. In another review, Jennings et al. [26] examined human pancreatic development and its developmental disorders, while Som and Miletich [27] reviewed the embryological processes involved in salivary gland development, paying particular attention to the important roles of and interactions between the epithelial cells, mesenchyme, and extracellular matrix.

The exocrine pancreas is arranged into acini (Figure 1), which produce digestive enzymes that play a major role in digestion. Pancreatic acini secrete pancreatic juice to complete the digestion of chyme in the duodenum. Pancreatic juice represents a complex mixture of water, salts, bicarbonate, and different digestive enzymes with substrate specificity: pancreatic amylase, pancreatic lipase, trypsin, chymotrypsin, carboxypeptidase, and nucleases (i.e., ribonuclease and deoxyribonuclease). The endocrine pancreas is composed of the islets of Langerhans, which harbor two main types of endocrine cell: *α*-cells that produce the hormone glucagon, which raises blood glucose levels, and *β*-cells that produce the hormone insulin, which lowers blood glucose levels. Thus, the endocrine pancreas controls glucose homeostasis.

The salivary glands produce exocrine and endocrine secretions that play a role in systemic health. Saliva, aside from its roles in digestion and as a taste perception aid, is important for oral cavity homeostasis, lubrification, immune defenses, and wound healing. This clear, serous fluid is mainly composed of water but also contains many important substances including electrolytes, mucus, salivary proteins and glycoproteins, antibacterial and antioxidant compounds, and various enzymes, in particular the digestive enzymes amylase and lingual lipase. Saliva is also an essential actor in oral food perception and acts as a solvent through which taste and aroma compounds are released into the oral cavity; its role also includes transport of taste substances to and protection of the taste receptor [28,29,30]. The endocrine salivary gland produces biologically active peptides and growth factors including EGF, nerve growth factor (NGF), transforming growth factor alpha (TGF-*α*), TGF-*β*, hepatocyte growth factor (HGF), insulin-like growth factor 1 (IGF-I), IGF-II, and basic fibroblast growth factor (bFGF). These factors play a key physiological role. They are released into the bloodstream to contribute to systemic health and play a role in salivary gland morphogenesis, wound healing, tissue regeneration, and immunomodulation [19,20], regulating several organ, tissue, and cellular functions including the pancreas, liver, lungs, stomach, intestine, and neutrophils. A decrease or loss of salivary endocrine function results in inappropriate or pathological responses of these tissues to various stressors [19]. Thus, the salivary glands participate in the regulation of body function and their dysfunction contributes to the development and maintenance of some pathological conditions [20]. In their absence, oral and systemic health are compromised, demonstrating that salivary secretions contribute to the regulation of many physiological processes.

### 2.2. Interaction between the Organs: The Role of the Growth Factor EGF

The salivary glands and the pancreas interact with one another via the action of growth factors. Several salivary gland factors (EGF and EGF analogues) appear to contribute to pancreatic tissue protection in response to injury by maintaining blood flow, preventing necrotic damage, and reducing activation of inflammatory cascades [19]. EGF is produced by glands in the gastrointestinal tract, especially the salivary and Brunner’s glands, the latter located in the small intestine. The excellent review by Marti et al. explored the biological effects of EGF with an emphasis on the gastrointestinal tract and liver [31]. EGF is a mitogenic growth factor that plays a fundamental role in morphogenesis, tissue regeneration, and ion transport. It also has anti-inflammatory and immunomodulatory effects through the regulation of cytokine and chemokine secretion [32]. Conversely, chronic pancreatitis of various etiologies, including alcoholic chronic pancreatitis [23] or autoimmune and idiopathic chronic pancreatitis [22], has been reported to have a secondary effect on the salivary glands, impairing their function in the course of the disease. In experimental studies of pancreatitis induced by caerulein, salivary gland removal resulted in an increase in plasma interleukin-1*β*, a proinflammatory cytokine that participates in acute and chronic inflammatory and autoimmune disorders. Exogenous EGF enhanced pancreatic recovery and prevented the pathological changes associated with salivary gland removal [19]. Salivary gland function is impaired in diabetes mellitus; the glandular dysfunction, resulting from a deficiency in salivary secretion of EGF, is associated with a chronic lymphocytic infiltrate, glandular disorganization, and destruction of the ductal cells, the source of salivary gland EGF [20].

### 2.3. Endocrine-Disrupting Compounds Perturb Organ Morphology and Function

The submandibular gland represents a good example of how endocrine-disrupting chemical (EDC) exposure may affect the morphogenesis, structure, and consequently function of an organ; exposure to EDCs is associated with a change in taste preference. Submandibular gland development is also a sensitive target of endocrine disruption that may have late-onset consequences. Kouidhi et al. [33] reported on the effects of genistein and vinclozolin, alone or in combination, on the submandibular salivary gland of immature female rats after in utero and lactational exposure. Gland morphogenesis and mRNA expression of endocrine growth factors were affected in all treated groups compared with untreated controls, and the effects were potentiated by the mixture. In particular, EGF, NGF, and TGF-*α* were repressed. The effects of the same exposure were further investigated with respect to the morphology and functions of submandibular salivary glands in immature and mature male rats after perinatal exposure [18]. Gestational and lactational exposure delayed submandibular salivary gland maturation in immature animals, as evidenced by the persistence of proacinar structures (embryonic acinar precursors) associated with an increase in sweet preference (enhanced saccharin intake and reduced water intake) compared to the structures and preferences of controls. Additionally, the mRNA expression of taste-related proteins (i.e., salivary proteins) in the submandibular exocrine salivary secretions, i.e., gustin (also called carbonic anhydrase 6), cystatin C, and mucin 10, which are all sex hormone-regulated proteins, were modified by all EDC treatments at immature stages, while the observed effects disappeared in mature animals, as did the differences in gland morphology and saccharin intake.

The enhanced mRNA expression of mucin 10 and gustin in rats treated at an immature stage may be another sign of developmental delay, as the proacini are high producers of mucin and are involved in sweet taste preference. Moreover, changes in the mRNA expression of growth factors (EGF, TGF-*α*, NGF) and steroid receptors (androgen receptor (AR), progesterone receptor (PR)) were also observed at different ages and following estrogenic and anti-androgenic chemical exposure. In addition to altering the glandular structure and secretory functions of submandibular salivary glands, an increase in sweet preference was observed, indicating delayed development, as the sweet taste is usually preferred by babies and juveniles. These findings suggest a link between taste preference, structure, and function of the submandibular glands in male rats and that endocrine disruptors could lead to early salivary gland dysfunction that may predict oral disease. Based on the above observations and discussion, it appears that an organ’s function can change following chemical exposure. Oral disease may manifest a long time after exposure to environmental chemicals as a consequence of deregulated expression of exocrine and endocrine secretions by the salivary glands. In addition, changes in taste preferences might be extrapolated to changes in eating habits and thus be related to nutrition. In addition, the morphology and function of pancreatic tissue may also be altered due to disturbed regulation by the salivary glands through growth factors.

The rodent pancreas has recently been shown to be a direct target of an EDC, which resulted in metabolic alterations. Yang et al. investigated the effects of perinatal exposure to nonylphenol (NP) on carbohydrate metabolism in male offspring rats [34] and found that pancreatic tissue NP concentrations in NP-exposed groups were four-fold higher than those in control groups. Morphological analysis showed that the NP-treated groups had a higher degree of inflammatory injury, edema, and focal necrosis in the pancreas. NP induced abnormal expression of glucokinase (GCK, downregulated) and uncoupling protein-2 (UCP-2, upregulated). This suggests that NP exposure could induce a glucose metabolism disorder in male F1 rats. Like bisphenol A (BPA), NP is considered an endocrine disruptor and acts as an estrogen mimic in affected organisms [35,36]. NP has been described as an inducer of numerous human health problems, with effects on metabolism (such as interfering with hypothalamic appetite control), pregnancy, and cancer. For example, NP has been shown to promote the proliferation of breast cancer cells due to ER*α* (estrogen receptor *α*) agonism in estrogen-dependent and -independent breast cancer cells. Diet appears to be the most significant source of NP exposure in humans, and microgram amounts of NP have also been found in the saliva of patients receiving dental sealants [37].

### 2.4. Endocrine-Disrupting Compounds Contribute to the Development of Type II Diabetes and Obesity

The discussion above highlights that diabetes mellitus could be considered a disease associated with the impairment of salivary gland function. Complementing this finding, Alonso-Magdalena et al. [38] recently highlighted a novel susceptibility for EDC exposure that may be important for the development of type II diabetes mellitus. They reported that bisphenol A (BPA) exposure during pregnancy has harmful long-term implications for the mother that manifest later in life. The study was conducted with pregnant mice and showed evidence of the impact of exposure to BPA on glucose metabolism, pancreatic *β*-cell function, and the regulation of *β*-cell mass. They focused on several cell cycle regulators, in particular cyclin D2 and CDK4 (cyclin-dependent kinase 4), which promote G phase to S phase progression and are known activators of *β*-cell cycling, proliferation, and mass. Moreover, BPA exposure in these mice altered the cell cycle inhibitor p16 and the key regulator of apoptosis p53. Among its numerous deleterious health effects, NP has been shown to both increase and decrease eating behavior by interfering with leptin signaling in the midbrain [39]. The hormone leptin signals the feeling of fullness after eating but, paradoxically, NP has both obesogenic and anti-obesogenic properties [39]. With respect to the former, NP has been shown to increase food intake by lowering the expression of anorexigenic neurons in the brain [40]. In addition, NP affects the expression of ghrelin, a circulating hormone produced by enteroendocrine cells of the gastrointestinal tract, especially the stomach. Ghrelin is often called a “hunger hormone” because it stimulates appetite and thus increase food intake [41]. Ghrelin also participates in the regulation of taste sensation and glucose metabolism. Ghrelin expression is positively regulated by estrogen signaling in the stomach, and it is also important in guiding the differentiation of stem cells into adipocytes. Acting as an estrogen mimic, prenatal and perinatal exposure to this NP has been shown to increase appetite and encourage the body to store fat later in life [42]. Insulin signaling in the liver of adult male rats has also been shown to be affected after long-term exposure to NP [43].

### 2.5. Four Interconnected Risk Factors for Pancreatic Cancer: Chemical Exposure, Diabetes, Obesity, and Eating Behavior

**Figure 2:**
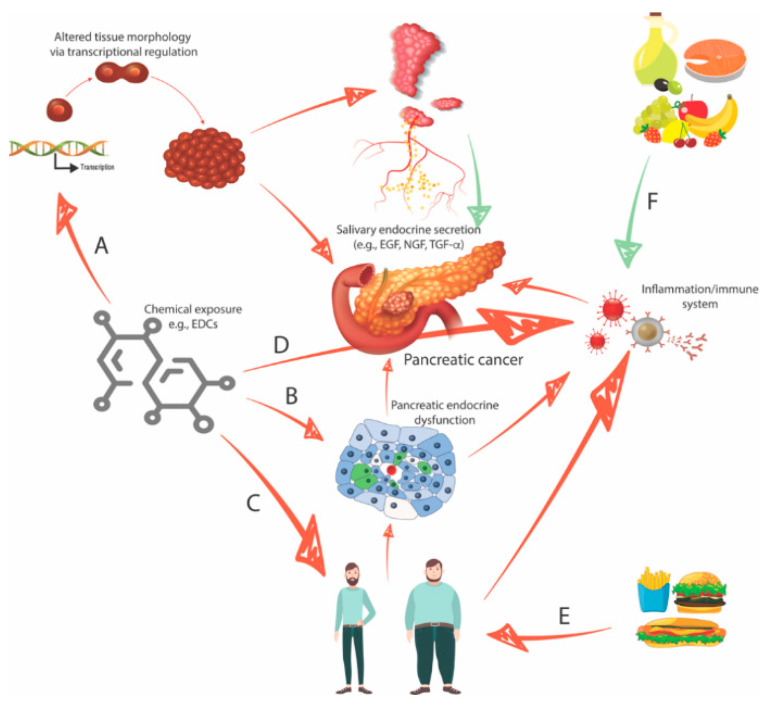
Four interconnected risk factors for pancreatic cancer: chemical exposure, obesity/diabetes, and eating behavior. (**A**) Chemical exposure, such as to -disrupting chemicals (EDCs), can affect both salivary gland and pancreatic morphology and function by altering transcriptional programs to either (i) directly affect pancreatic cell growth and function or (ii) alter salivary gland endocrine secretions (e.g., EGF) to have a distant effect on pancreatic function. (**B**) EDC exposure may have a direct diabetogenic effect on pancreatic *α*-cells and *β*-cells, promoting insulin resistance, diabetes, and/or predisposition to cancer or by inducing a pro-inflammatory state that is carcinogenic. (**C**) EDC exposure may be obesogenic by directly stimulating appetite via hormone pathways, promoting insulin resistance and diabetes, and/or predisposing to cancer or by inducing a pro-inflammatory state that is carcinogenic. (**D**) The immune system constitutes another target of EDC action, as for natural hormones. (**E**,**F**) The effects of diet can be positive or negative, depending on the type of food, with high-fat, carbohydrate-rich foods promoting obesity and its consequent effects, and the so-called Mediterranean diet having anti-inflammatory effects and contributing to boosting the immune system that might reduce the subsequent pancreatic cancer risk (green arrow in F).

Taken together, chemical exposure, diabetes, obesity, and eating behavior can be considered interconnected risk factors for pancreatic cancer. Figure 2 proposes several hypotheses to explain how pancreatic carcinogenesis may occur through the interplay between the organs, the role of environmental factors (diet and chemical exposure), and putative non-genomic alterations or mutational events that primarily drive PDAC genesis.

There is epidemiological evidence that excess weight and obesity increase the risk of several types of cancer including PDAC. Obesity is not only linked to cancer initiation and progression but also to metabolic and other obesity-related diseases, e.g., diabetes, another risk factor for pancreatic cancer [44]. Adipose tissue has a very strong endocrine function, secreting various adipokines involved in cancer development, invasion, and metastasis and insulin resistance. Leptin, IL-6, and TNF-*α* are proinflammatory cytokines produced by adipose tissue and that are also increased in cancer, while adiponectin protects against tumorigenesis and its serum levels are usually decreased in cancer patients. Cancer patients also show higher baseline levels of C-reactive protein, an inflammatory marker used in routine clinical practice. Obese and overweight individuals have high circulating leptin levels but exhibit leptin resistance, therefore contributing to failed food intake control mechanisms. High leptin levels can induce cancer cell proliferation and thus provide a link between obesity and cancer progression. In fact, leptin signaling is linked to the development of several cancer types, including pancreatic cancer, through different mechanisms including the production of inflammatory factors (IL-1, IL-6, and TNF-*α*), which have been shown to promote tumor invasion and metastasis [1]. Obesity is also linked to chemoresistance, with a high Body Mass Index (BMI) being associated with a decreased response to chemotherapy. The review by Cascetta et al. [45] summarizes the molecular mechanisms linking pancreatic cancer and obesity through chemoresistance in high BMI patients. Obesity is thought to be associated with the failure of antiangiogenic therapy, and obesity can increase tumor inflammation.

IL-6 has local effects on different cell types, inducing a state of inflammation. Anti-vascular endothelial growth factor (VEGF) therapy has failed to improve survival in patients with some types of cancer such as metastatic colon or kidney cancer, whereas obesity was recently shown to have an effect on anti-VEGF responses in breast cancer patients [46]. Their findings indicated that obesity was associated with resistance to anti-VEGF therapy via the production of inflammatory and angiogenic factors. IL-6 blockade abrogated obesity-induced resistance to anti-VEGF therapy at primary and metastatic sites.

Diabetes is a complex metabolic disorder of glucose control. Insulin is produced by pancreatic *β*-cells, and it allows glucose to enter cells for energy production. Insulin also acts as a chemical messenger that instructs the liver to store glucose in the form of glycogen. Without insulin, glucose circulates and excess glucose is excreted into the urine. Type 1 diabetes (insulin-dependent diabetes mellitus (IDDM)) mostly occurs in children and represents a complete failure of pancreatic insulin production; type 1 diabetics must therefore receive replacement insulin by injection. Type 1 diabetes is thought to arise either as (1) an inherited cause, or (2) through an auto-immune process characterized by *β*-cell destruction. Although there may be an environmental trigger for the disease, there are no known ways to prevent type 1 diabetes. Since perinatal exposure to NP morphologically alters rat pancreatic tissues [34], we can hypothesize that this can lead to organ dysfunction and thus disturb or ablate exocrine and/or endocrine secretions.

Type 2 diabetes, or non-insulin-dependent diabetes mellitus (NIDDM), mainly occurs in adults but increasingly so in children. Type 2 diabetes is associated with obesity; while type 2 diabetics usually produce enough insulin, their cells cannot use it effectively, a process known as insulin resistance. A failure of glucose absorbance increases circulating glucose levels, which, when below the threshold indicating diabetes, is referred to as prediabetes and indicates people who are likely to develop type 2 diabetes or with insulin resistance. Prediabetics secrete more insulin to maintain normal blood sugar levels, but over time the pancreas cannot maintain this extra insulin release, and type 2 diabetes develops in the absence of control measures. In addition, high insulin levels drive obesity and further exacerbate insulin resistance, resulting in a vicious cycle. As the normal balance between calorie intake and insulin secretion is disrupted in diabetes, the effectiveness of therapeutic fasting to reverse insulin resistance has been demonstrated [47]. Insulin secretion is stimulated to different degrees by carbohydrates, proteins, and fat. Refined carbohydrate-rich foods are insulin-stimulating and therefore more obesogenic than non-insulin-stimulating foods, even if they are calorie equivalent. This phenomenon highlights the importance of selecting a dietary balance that minimizes insulin secretion, such as a Mediterranean diet.

An association between cancer and diabetes was identified in the 1960s in population-based studies [48]. In addition, cancer and diabetes have both been described as chronic inflammatory diseases [49,50,51]. Behaviors leading to anti-inflammatory reactions are beneficial to health, especially diet and exercise, both of which can stimulate the immune system to produce an anti-inflammatory cellular response. Numerous experimental studies have shown that foods or beverages may have pro- or anti-inflammatory effects, with refined carbohydrates, red meat, and fried foods being particularly pro-inflammatory. Therefore, certain foods associated with excessive inflammation are also associated with insulin-stimulating foods, fat storage, and obesity. By contrast, anti-inflammatory foods include tomatoes, olive oil, green leafy vegetables, fruits, oily fish such as salmon, honey, nuts, and coffee, which contains polyphenols and other anti-inflammatory compounds.

Therefore, blood sugar levels are elevated as a consequence of diabetes, which may itself be caused in different ways including exposure to chemicals such as endocrine disruptors, which affect insulin production. Other metabolites resulting from glucose metabolism are as a consequence modified and may then affect their targets, which may be epigenetic enzymes. The epigenome and gene expression may then be disturbed as a consequence of a cascading reaction. There has been evidence implicating EDC exposure in the etiology of type 2 diabetes and obesity since 2008. However, the specific EDCs acting as risk factors for obesity, diabetes, or both have been difficult to define due to the close relationships between these pathologies. Some EDCs act as obesogens, while others act as diabetogens. For a comprehensive overview of the literature on how EDCs are implicated in the etiology of obesity and type 2 diabetes, readers are referred to Gore et al. [52].

Finally, the immune system plays a role in protecting against tumors but also constitutes another target of EDC action, as for natural hormones [53], including but not limited to immunosuppressive effects. The aquatic environment is known to be affected by EDCs, in particular estrogen mimics. In addition to the reproductive system, EDCs may affect a variety of physiological systems, including the immune systems of fish. First, estrogen receptors are expressed in fish immune organs, and estrogen-related molecules in fish are immunomodulatory and increase pathogen susceptibility in fish [54]. Overall, fish immune systems appear to be weakened by estrogen exposure; this has direct implications for humans. Indeed, EDCs have been shown to interfere with cytokine and immunoglobulin synthesis and also affect immune cell activation and survival. Briefly, EDCs modulate the human immune system by decreasing immunity, inducing allergic diseases and inflammation, and inducing autoimmune diseases [55]. Faulty hormonal imprinting is an epigenetic process caused by the binding of natural hormone-related molecules such as EDCs to target hormone receptors. This can contribute to disease pathogenesis in any period of life but has particularly important consequences in the perinatal period through transgenerational inheritance via epigenetic mechanisms. Indeed, faulty imprinting-provoked pathological alterations that manifest later in adulthood have been described, not limited to the immune system. Consistent with the findings from others, Csaba [56] reported an increased susceptibility to inflammatory responses and weakened protective and regulatory immune functions in adult offspring after perinatal exposure to EDCs.

## 3. Urgent Need for Early Diagnosis: Identification of Salivary Biomarkers and Development of Screening Tools

Early detection of PDAC is essential in order to optimize management and to improve outcomes. With regard to early diagnosis, acute pancreatitis has been shown in some studies to be associated with increased pancreatic cancer risk. Acute pancreatitis is an inflammatory disease of the pancreas leading to its fibrotic destruction. For instance, a recent Danish nationwide, matched cohort study showed that patients hospitalized with acute pancreatitis had an increased risk of developing pancreatic cancer compared with the general population after five years of follow-up [57]. Chronic pancreatitis also increases the risk of pancreatic cancer, but the association weakens with long-term follow-up [58]. For a comprehensive review of the current diagnosis and management of chronic pancreatitis, see [59]. Diabetes is also a known risk factor for PDAC but can also be an initial symptom of the disease [9,60]. Therefore, patients with new-onset diabetes may benefit from imaging for PDAC [3].

Human saliva has emerged as a valuable, non-invasive, and easy to collect diagnostic tool for the detection of various local and systemic diseases. Therefore, saliva is a particularly attractive fluid for biomarker research and development, health and disease surveillance, and personalized medicine. A number of studies have demonstrated that saliva is useful for the detection and diagnosis of various diseases including malignancies (including pancreatic cancer), human immunodeficiency virus, cardiac disease, autoimmune diseases, oral diseases [61,62,63,64,65,66,67,68], some of which have been associated with salivary antioxidant system dysfunction [23] and oxidative damage to proteins and lipids [64,65,66]. Diseases can be reflected in and monitored by salivary biomarker signatures [62,63,64,66,68,69], and a wide range of salivary biomarkers have been validated [68]. If well validated, these biomarkers could help clinicians to predict future disease occurrence and prompt more intensive monitoring for these diseases to detect them at an early stage, prompt early management, and thereby improve outcomes.

The term “salivaomics” was introduced in 2008 to describe the five diagnostic components and “omics” constituents of saliva: proteins, mRNAs, micro-(mi) RNAs, metabolic compounds, and microbes [70]. For an excellent review of salivaomics, see [71], which particularly focuses on salivary nucleic acids and proteins as potential biomarkers for the detection and monitoring of several cancer types. Saliva extracellular RNAs (exRNAs) are abundant and stable species that have become a useful source of biomarkers of local and systemic diseases including but not limited to pancreatic cancer [72,73]. RNA-sequencing (RNA-seq) is now a widely used high-throughput approach to profile exRNAs; however, selection of the optimal methods for RNA isolation and cDNA library construction need careful protocol development; for a review of current methods for salivary exRNA studies, see [73]. Of the different transcripts, salivary miRNAs seem to be particularly promising molecules for cancer diagnosis [74]. In particular, a number of pancreatic cancer-specific miRNAs have been identified for pancreatic cancer [74,75]. Among these, the salivary miRNAs miR-3679-5p and miR-940 appear to have good discriminatory power for differentiating pancreatic cancer from healthy controls and are potential pancreatic cancer biomarkers [76]. The salivary microbiome has also been investigated as a possible source of biomarkers, with microbiome variability shown to be associated with early resectable pancreatic cancer [77].

How can salivary biomarkers reflect disease states elsewhere in the body, and how and why do diseases distant from the oral cavity lead to the development of discriminatory biomarkers in saliva? One possible explanation is salivary exosomes, nanoscale extracellular vesicles that are secreted by most cells, which have been identified as stable and clinically relevant species for cancer detection [69,71]. A working hypothesis is that exosomes can shuttle tumor-specific contents to different parts of the body including the salivary glands, leading to the appearance of disease-discriminatory markers in saliva [70]. Indeed, the role of exosomes in generating specific biomarkers in the saliva has been demonstrated in vivo [78]. Lau et al. demonstrated that inhibition of pancreatic cancer-derived exosomes in mice altered the disease-specific transcriptomic biomarker profile of saliva, supporting that pancreatic tumor-derived exosomes can be involved in the development of discriminatory salivary biomarkers. These transcriptional changes in the salivary glands resulted from direct contact of pancreatic tumor-derived exosomes with the salivary glands. This explains how tumor-derived exosomes relay information to generate specific salivary biomarkers. These recent advances suggest that salivary diagnostics have huge potential translational and clinical application, including for personalized medicine.

Defined genomic alterations, such as *BRCA1* mutations, could predispose to early salivary gland dysfunction and decrease concentrations of salivary proteins [66]. Since other environmental factors including diet, tobacco smoking, and alcohol are also associated with an increased risk of pancreatic cancer, behavior modification also remains an important strategy in preventing the disease [9].

## 4. Conclusions

Pancreatic cancer is a complex disease driven by intrinsic and extrinsic factors. There have been clinical and preclinical advances in the management of pancreatic cancer over the last decade, but further improvements in knowledge of pancreatic cancer biology are required to underpin clinical efforts to develop new therapeutic strategies. Early diagnosis remains an essential priority, as PDAC usually presents late and therefore at an advanced stage. New high-throughput technologies have led to the identification and validation of numerous biomarkers. The saliva is a particularly attractive source of non-invasive biomarkers and diagnostic tools. Nevertheless, there remains a need to identify efficient and specific pancreatic cancer biomarkers for early disease detection and with clinical application. Pancreatic cancer risk is increased by several factors including tobacco and alcohol consumption and obesity, which are modifiable. Therefore, preventive measures such as a balanced diet, reduced consumption of red meat and alcohol, stopping smoking, and regular physical activity are essential. Chemical pollutants such as endocrine disruptors may be another avoidable risk factor, especially in pregnant women. These pollutants may also induce type 2 diabetes, which is associated with fat storage and obesity, perhaps as a consequence of tissue damage (particularly to insulin-secreting *β*-cells) and consequent pancreatic dysfunction. Salivary glands may also be targeted by any toxic chemical that leads to the destruction of ductal cells or disrupts their function, thus impacting endocrine secretion such as EGF and pancreatic regulation. Chemical exposure could also disrupt pancreatic morphogenesis through the dysregulation of the expression of cell cycle-, differentiation-, and metabolism-related genes and favor (or predispose to) carcinogenesis. In addition, these processes may be associated with non-genomic alterations or mutational events that primarily drive PDAC genesis in an inflammatory context; future efforts should explore ways to avoid inflammatory processes and to promote anti-inflammatory reactions. Finally, this review highlights the important role of the salivary glands and environmental factors on pancreatic homeostatic mechanisms and normal regulation through salivary gland dysfunction.

## Abbreviations

PDAC	Pancreatic ductal adenocarcinoma
USA	United States of America
FOLFIRINOX	Folinic acid, 5-fluorouracil, irinotecan, and oxaliplatin
EGF	Epidermal growth factor
NGF	Nerve growth factor
TGF-α	Transforming growth factor alpha
TGF-β	Transforming growth factor bêta
HGF	Hepatocyte growth factor
IGF-I	Insulin-like growth factor 1
IGF-II	Insulin-like growth factor 2
bFGF	Basic fibroblast growth factor
EDC	Endocrine-disrupting chemical
AR	Androgen receptor
PR	Progesterone receptor
NP	Nonylphenol
BPA	Bisphenol A
ERα	Estrogen receptor alpha
CDK4	Cyclin-dependent kinase 4
IL-6	Interleukin 6
IL-1	Interleukin 1
BMI	Body mass index
VEGF	Vascular endothelial growth factor
IDDM	Insulin-dependent diabetes mellitus
NIDDM	Non-insulin-dependent diabetes mellitus
